# Maternal Exposure to Nitrogen Dioxide during Pregnancy and Offspring Birth Weight: Comparison of Two Exposure Models

**DOI:** 10.1289/ehp.0901509

**Published:** 2010-05-14

**Authors:** Johanna Lepeule, Fabrice Caïni, Sébastien Bottagisi, Julien Galineau, Agnès Hulin, Nathalie Marquis, Aline Bohet, Valérie Siroux, Monique Kaminski, Marie-Aline Charles, Rémy Slama, the EDEN Mother–Child Cohort Study Group

**Affiliations:** 1 INSERM, Avenir Team “Environmental Epidemiology Applied to Fecundity and Reproduction,” Institut Albert Bonniot, Grenoble, France; 2 University J. Fourier Grenoble, Grenoble, France; 3 Atmo Poitou-Charentes, Perigny, France; 4 Airlor, Vandoeuvre les Nancy, France; 5 INSERM, U1018, Centre de Recherche en Epidémiologie et Santé des Populations, Team “Epidemiology of Reproduction and Child Development,” Le Kremlin-Bicêtre, France; 6 University of Paris-Sud 11, UMR 1018, Le Kremlin Bicêtre, France; 7 INSERM, Team “Epidemiology of Cancer and Severe Diseases,” Institut Albert Bonniot, Grenoble, France; 8 UMR 953, Institut Fédératif de Recherche 69, Epidemiological Research Unit on Perinatal and Women’s and Children’s Health, Villejuif, France; 9 UPMC, Paris, France; 10 INSERM, U1018, Centre de Recherche en Epidemiologie et Santé des Populations, Team “Epidemiology of Obesity, Diabetes and Renal Disease over the Life Course,” Villejuif, France

**Keywords:** atmospheric pollution, birth weight, cohort, exposure modeling, geostatistical, measurement error, monitoring station, nitrogen dioxide, spatial variation, temporal variation

## Abstract

**Background:**

Studies of the effects of air pollutants on birth weight often assess exposure with networks of permanent air quality monitoring stations (AQMSs), which have a poor spatial resolution.

**Objective:**

We aimed to compare the exposure model based on the nearest AQMS and a temporally adjusted geostatistical (TAG) model with a finer spatial resolution, for use in pregnancy studies.

**Methods:**

The AQMS and TAG exposure models were implemented in two areas surrounding medium-size cities in which 776 pregnant women were followed as part of the EDEN mother–child cohort. The exposure models were compared in terms of estimated nitrogen dioxide (NO_2_) levels and of their association with birth weight.

**Results:**

The correlations between the two estimates of exposure during the first trimester of pregnancy were *r* = 0.67, 0.70, and 0.83 for women living within 5, 2, and 1 km of an AQMS, respectively. Exposure patterns displayed greater spatial than temporal variations. Exposure during the first trimester of pregnancy was most strongly associated with birth weight for women living < 2 km away from an AQMS: a 10-μg/m^3^ increase in NO_2_ exposure was associated with an adjusted difference in birth weight of −37 g [95% confidence interval (CI), −75 to 1 g] for the nearest-AQMS model and of −51 g (95% CI, −128 to 26 g) for the TAG model. The association was less strong (higher *p*-value) for women living within 5 or 1 km of an AQMS.

**Conclusions:**

The two exposure models tended to give consistent results in terms of association with birth weight, despite the moderate concordance between exposure estimates.

Several epidemiologic studies have reported associations between maternal exposure to nitrogen dioxide (NO_2_) during pregnancy and fetal growth assessed by birth weight, taking into account gestational duration (e.g., Bell et al. 2007; Liu et al. 2007; Ritz and Wilhelm 2008; Slama et al. 2008; Wilhelm and Ritz 2003). Various approaches may be used to estimate exposure, from the use of biomarkers of exposure to personal dosimeters and environmental models. Most previous studies have been based on measurements from permanent air quality monitoring stations (AQMSs), using data from the AQMS closest to the subject’s home address or interpolating data for neighboring monitors, for which measurements are averaged over the entire pregnancy or over each trimester of pregnancy. This approach has the advantage of making use of readily available exposure data, being simple to implement and, because pollutants are assessed on an hourly or at least weekly basis, being highly flexible in terms of the temporal exposure window considered. However, the spatial density of AQMS networks is generally low, and studies have shown that the data provided by permanent AQMSs are representative only of air pollution levels in the close vicinity of the station (Lebret et al. 2000). Studies based on AQMS measurements assume that air pollution levels are homogeneous within a buffer of several kilometers around each monitor or, at least, that exposure misclassification introduces no major bias into the estimated exposure–response relationship. However, studies based on the simultaneous use of several exposure models have demonstrated that the amplitude of the measurement error may be large (Nerriere et al. 2005; Nethery et al. 2008; Sarnat et al. 2005). Moreover, at least for respiratory or cardiovascular outcomes, measurement error may have a large impact on the exposure–response relationship (Miller et al. 2007; Van Roosbroeck et al. 2008). This issue has very little been studied in the context of reproductive outcomes (Brauer et al. 2008).

We aimed to compare the exposure model based on the nearest AQMS and a temporally adjusted geostatistical (TAG) model based on measurement campaigns with a fine spatial resolution, and also focusing on background pollution, in the context of a mother–child cohort. We compared these models in terms of estimated NO_2_ levels and the estimated association between NO_2_ levels and birth weight.

## Materials and Methods

### Study population and data collection

This study was conducted in a subgroup of the French EDEN (study of pre- and early postnatal determinants of the child’s development and health) mother–child cohort. Pregnant women at < 26 weeks of gestation were recruited from the maternity wards of Poitiers and Nancy university hospitals (France) between September 2003 and January 2006. Gestational age was assessed from the date of the last menstrual period (Slama et al. 2009). Exclusion criteria were a personal history of diabetes, multiple pregnancy, intention to deliver outside the university hospital or to move out of the study region within the next 3 years, and an inability to speak and read French. The birth weights of the infants were extracted from the maternity records. Information on maternal active and passive smoking, height, weight, and educational level were collected by interview between 24 and 28 weeks of gestation, and by questionnaire after birth. The study was approved by the relevant ethical committees (Comité Consultatif pour la Protection des Personnes dans la Recherche Biomédicale, Le Kremlin-Bicêtre University Hospital, and Commission Nationale de l’Informatique et des Libertés), and all participating women gave informed written consent for their own participation and that of their children. More details of this study can be found elsewhere (Drouillet et al. 2009; Slama et al. 2009; Yazbeck et al. 2009).

### Exposure to NO_2_

**Figure 1:**
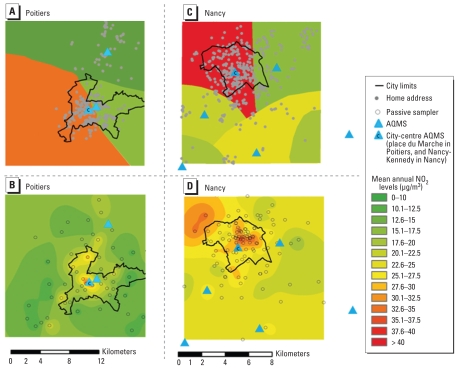
Mean annual NO_2_ levels estimated by the nearest-AQMS model in Poitiers (*A*) and Nancy areas (*C*) and by the TAG model in Poitiers (*B*) and Nancy areas (*D*).

We restricted the cohort to pregnant women living in two areas, one of 165 km^2^ around Nancy and the other of 315 km^2^ around Poitiers, in which air quality measurement campaigns have been conducted. We then further restricted the study area to the immediate vicinity of an AQMS, focusing on circular buffers with a radius of 5, 2, and 1 km around each AQMS (Figure 1B,D). The detailed addresses of all women were geocoded in ArcGIS (version 9.3; ESRI, Redlands, CA, USA). For both models, changes of home address between inclusion and delivery were taken into account by calculating time-weighted means of exposure over the relevant time windows [whole pregnancy, and each trimester (92 days per trimester if no delivery) of pregnancy].

### Nearest-AQMS model (model 1)

We obtained air pollution data from the Airlor (Nancy) and Atmo-Poitou-Charentes (Atmo-PC)(Poitiers) AQMS networks. All permanent AQMS measuring NO_2_ concentrations during the study period and located within 2.5 km of the limits of the study areas were considered (three in the Poitiers area and six in the Nancy area) (Figure 1A,C), excluding those labeled as traffic (i.e., located < 5 m from a road with traffic levels of > 10,000 vehicles/day) (Agence de l’Environnement et de la Maîtrise de l’Energie 2002) or industrial stations. For each woman *i*, hourly measures of NO_2_ concentration by the AQMS *j* closest to her home address were averaged over each time window Δ*^i^_t_* considered (noted Δ*_t_* for convenience), to obtain our exposure estimate E1*^i^_j_*_,Δ_*_t_*.

### TAG model (model 2)

NO_2_ measurement campaigns with a Palmes diffusive sampler (Palmes et al. 1976) were conducted in the urban and periurban areas of both cities. The diffusive samplers were located so as to give measurements of background pollution in each area (61 locations in the Poitiers area, 98 locations in the Nancy area). The campaigns lasted 14 days (Poitiers) or 10–15 days (Nancy) and were repeated throughout the year to capture seasonal variations. Nine campaigns were performed in 2005 in the Poitiers area, and 10 were performed in 2002 in the Nancy area (Airlor 2004; Atmo-PC 2007). In each area, for each passive sampler, the AQMS giving the measurements most strongly correlated with the measurements of the passive sampler during campaigns was used to estimate mean annual concentration at each measurement location. These estimated annual concentrations were smoothed over the whole area with kriging techniques (Chilès and Delfiner 1999) on a 50 × 50 m grid, with Isatis software version 6.06 (Géovariances, Fontainebleau, France) (Figure 1B,D). This corresponded to our estimate of *C^i^*_yearly_, the mean NO_2_ concentration at the home address, for the year 2005 in Poitiers and 2002 in Nancy (spatial component of the model).

The estimated annual NO_2_ concentrations were then combined with time-specific measurements from the permanent AQMS to capture temporal variations in concentrations. This approach has previously been used in the context of land use regression (LUR) models (Slama et al. 2007). The hourly NO_2_ measures of all AQMSs from the area were averaged over each time window Δ*_t_* considered (*S^i^*_all, Δ_*_t_*) and also over the year in which the measurement campaign was performed (*S*_all, yearly_). The ratio


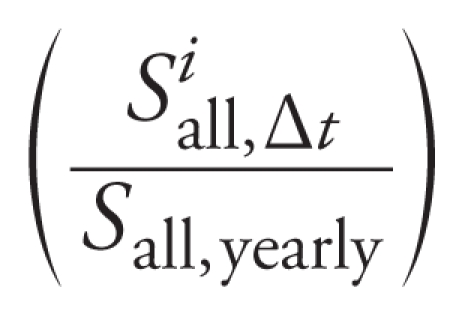


was the temporal component of the model. The temporally adjusted estimate of NO_2_ exposure *E*2*^i^*_Δ_*_t_* for woman *i* was the product of the spatial and temporal components, or


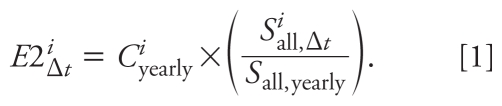


### Statistical analyses

For each model, we assessed the relative contribution of spatial (or temporal) variations in exposure contrasts by Pearson’s correlation coefficient between the exposure estimate and its spatial (or temporal) component. We also carried out variance decomposition. The nearest-AQMS model could be broken down as


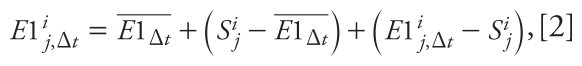


with 
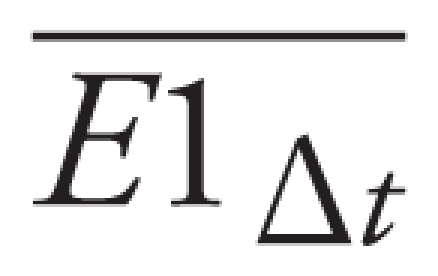
 the mean level of exposure of all women during the time window Δ*_t_*, and *S^i^_j_* the NO_2_ concentration at AQMS *j* averaged over the entire study period, so as to obtain a spatial component 
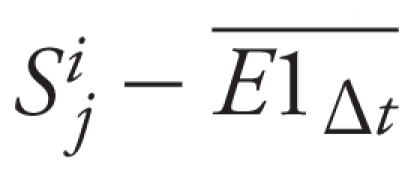
 dependent solely on the address of the woman. This corresponded to our estimate of the spatial component of the AQMS model; *E*1*^i^_j_*_, Δ_*_t_* – *S^i^_j_* corresponded to our estimate of the temporal component of the model. The TAG model was log-transformed and expressed as


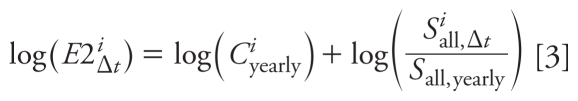


for the variance analysis. These analyses were restricted to women who did not change address during pregnancy.

For comparison of the exposure estimates generated by each model, exposure estimates for the two models were compared by Kruskal–Wallis rank tests and by calculating correlation coefficients (*r*). The distributions of the exposures estimated by the nearest-AQMS model and by the TAG model were plotted as a function of the AQMS closest to woman’s home address, with and without excluding the AQMS located in the city center. We also assessed the concordance between the estimates generated by the two models, classified into tertiles, by determining percentage concordance and the κ coefficient. Bland–Altman plots were used to estimate the magnitude of the systematic error between the two exposure models (Bland and Altman 1986).

For exposure–response relationships, we studied the relationship between birth weight and NO_2_ exposure during each exposure window in linear regression models taking into account gestational age and adjustment factors. Linear trend tests were performed with a categorical variable, the value of which corresponded to the category-specific median NO_2_ concentration. The adjustment factors were selected on the basis of *a priori* knowledge (Rothman et al. 2008). We adjusted for active and passive smoking during the second trimester of pregnancy, because these factors were more strongly associated with birth weight than were exposures during the first trimester, the third trimester, or all three trimesters combined. We also adjusted for sex of the newborn, maternal height (as a continuous variable), prepregnancy weight (broken stick model with a knot at 60 kg), birth order, maternal age at end of education, center, and trimester of pregnancy. Statistical analyses were carried out with STATA statistical software (Stata SE version 10.1; StataCorp LP, College Station, TX, USA). Analyses were repeated for the three buffers considered (< 5, 2, or 1 km from an AQMS).

## Results

### Population

Of the 1,893 women from the cohort with a known offspring birth weight, 776 lived in the study area, < 5 km from an AQMS, during at least one trimester of pregnancy (431 and 158 women lived within 2 and 1 km of an AQMS, respectively). Mean birth weight was 3,284 g (25, 50, 75th percentiles: 3,005, 3,310, 3,620 g). Table 1 shows the characteristics of the study population.

### Exposure to air pollutants

Estimates of exposure to NO_2_ were higher in Nancy than in Poitiers, whatever the exposure model and exposure window considered (Figure 1, Tables 1 and 2). The nearest-AQMS model estimate during pregnancy was more strongly correlated with the spatial component of the TAG model (*r* = 0.61, 0.68, and 0.84, for the 5-, 2-, and 1-km buffers, respectively) than with its temporal component (*r* = 0.35, 0.35, and 0.45, respectively). For both models, exposure estimates throughout pregnancy were subject to strong spatial variation (accounting for > 90% of the variance of exposure; Table 3). Temporal variations made a greater contribution to total variation when we considered trimester-specific windows but remained smaller than spatial variations for the nearest-AQMS model (72–84% for spatial variation and 20–25% for temporal variation), whereas the contributions of the spatial and temporal variation components were similar for the TAG model (43–61% for spatial variation and 44–57% for temporal variation; Table 3). The buffer around the AQMS studied had no major effect on the relative contributions of spatial and temporal components of variation.

**Figure 2:**
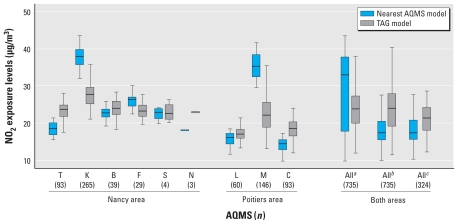
Box plots (25th, 50th, and 75th percentiles) of NO_2_ exposure levels during the whole pregnancy as estimated by the nearest-AQMS model and by the TAG model, according to the AQMS closest to the residential address. The population was restricted to 735 women living < 5 km away from an AQMS without change of assigned station during pregnancy. Abbreviations: T, Tomblaine; K, Nancy-Kennedy; B, Nancy-Brabois; F, Fléville; S, St Nicolas de Port; N, Neuves-Maison; L, Les couronneries; M, Place du marché; C, Chasseneuil. Stations were located in the periurban area. K (Nancy) and M (Poitiers) are stations located in the city center. ^a^Exposures estimated taking into account all AQMS. ^b^Exposures estimated taking into account all AQMS except K and M (city-center stations); for subjects initially assigned to one of these stations, the closest station has been replaced by the second AQMS nearest to the home address located outside the city center and < 5 km away from the home address, if any. ^c^Exposures were estimated taking into account all AQMS except K and M, with all women for whom K or M was the closest station excluded from the analysis.

The levels and range of NO_2_ concentrations estimated by the nearest-AQMS model were greater than those estimated by the TAG model (Table 2). Bland–Altman plots [see Supplemental Material, Figure 1 (doi:10.1289/ehp.0901509)] showed that the difference between the two models increased with mean exposure estimates. This pattern was principally due to between-model differences for women living in the city centers (mean NO_2_ concentrations estimated by the nearest-AQMS model were higher and ranges were narrower than for the TAG model), rather than in the periurban areas. Indeed, the exposure distributions for the two models became more similar when we did not take into account city-center AQMS measurements (Figure 2). All this indicates that the overestimation of NO_2_ exposure levels by the AQMS model with respect to the TAG model mainly concerned the women who were also the most exposed with the TAG model.

The correlation and concordance (κ) between the two exposure models were fair (0.40–0.74) when we considered all the women living within 5 km of an AQMS [Table 2; see also Supplemental Material, Figure 2 (doi:10.1289/ehp.0901509)] but were stronger if we restricted the study population to women living within 2 (0.37–0.79) or 1 km (0.59–0.87) of an AQMS. The correlation and concordance between the two exposure models also differed between the areas (Nancy/Poitiers) and between the city center and suburban areas [see Supplemental Material, Figure 2 (doi:10.1289/ehp.0901509)].

### Associations between air pollutants and fetal growth

**Figure 3:**
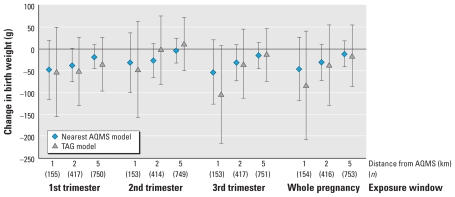
Change in mean birth weight (g) for a 10-μg/m^3^ increase in NO_2_ during pregnancy, as a function of the size of the buffer considered around each AQMS, adjusted for factors as described in “Materials and Methods.” Error bars indicate 95% CIs.

The patterns of association with birth weight identified were similar for the two exposure models, in terms of estimates of adjusted effects and confidence intervals (CIs), although these associations were stronger for the nearest-AQMS model [Figure 3; see also Supplemental Material, Table 1 (doi:10.1289/ehp.0901509)]. The first and third trimesters of pregnancy corresponded to the exposure windows most clearly associated with effects on birth weight, for both exposure models. For women living < 2 km from an AQMS, a 10-μg/m^3^ increase in NO_2_ concentration during the first trimester of pregnancy was associated with an adjusted change in mean birth weight of −37 g (95% CI, −75 to 1 g) for the nearest-AQMS model and of −51 g (95% CI, −128 to 26 g) for the TAG model. We obtained qualitatively similar results when we coded exposures in tertiles [see Supplemental Material, Table 1 (doi:10.1289/ehp.0901509)]. For the AQMS model, the parameter quantifying the association between NO_2_ exposure and birth weight approached zero as buffer size increased. We obtained similar results if we made no adjustment for city center (data not shown).

## Discussion

Our study is one of the first to describe associations between NO_2_ exposure assessed with a TAG model and birth weight, and to compare this model with the more commonly used approach based on permanent AQMSs. We compared models in terms of both exposure estimates and association with birth weight. The nearest-AQMS model was influenced by the location of monitors. Variations in exposure were mostly attributable to spatial rather than temporal variations in both models, with temporal variation making a larger overall contribution to total variation in the TAG model than in the nearest-AQMS model. The concordance between NO_2_ exposure estimates with the two models was fair when we considered the 5-km buffer. This concordance was stronger if we restricted the analysis to women living closer (< 2 km and, more clearly, < 1 km) to an AQMS. When we coded exposure as a continuous term, associations with birth weight for the TAG model were consistent with those obtained in analyses based on exposure estimated from the nearest-AQMS model, for the various buffers around AQMS and exposure windows.

The TAG model is thought to have a better spatial resolution than the nearest-AQMS model, because of the use of data from fine measurement campaigns, with no loss of temporal resolution, because we seasonalized TAG exposure estimates on the basis of AQMS measurements. The stronger contribution of the spatial component in the nearest-AQMS model than in the TAG model may at first glance appear counterintuitive, because the AQMS model could be considered to be essentially based on temporal variations. However, this finding may be accounted for by the considerable variation of the concentrations obtained with different AQMSs, some of which (in the city center) were influenced by traffic, despite meeting the criteria for background stations. This illustrates the extent to which the nearest-AQMS estimates depend on the location of the monitors, and the need for exposure models with a finer spatial resolution in studies with medium- or long-term exposure windows (3–9 months in our study). Because passive samplers were located at background sites less affected by traffic, the TAG approach led to a more purely background model than did the AQMS approach. The higher concentrations estimated by the nearest-AQMS model than by the TAG model (Table 2) may be accounted for by this feature. The TAG model may also smooth extreme exposure values, leading to an underestimation of the role of spatial variation.

One possible limitation of the TAG model stems from the approach used to seasonalize this model, in which we assumed that spatial differences in exposure remained constant over time. This assumption was found to be reasonable for a LUR model developed in Rome (Porta et al. 2009) but may not hold in other areas with different characteristics.

Several studies have evaluated the performance of AQMS for estimating exposure to air pollutants. Nerriere et al. (2005), Nethery et al. (2008), and Sarnat et al. (2005) reported poor concordance between AQMS estimates and personal monitoring data, which is not surprising because personal exposure is not expected to strictly correspond to background levels of air pollution at the home address. Marshall et al. (2008) reported correlations and κ-coefficients for estimates from the nearest-AQMS model (within 10 km) and estimates stemming from either an LUR (*r* = 0.61, κ = 0.42) or a dispersion model (*r* = 0.37, κ = 0.22). The concordance obtained with the LUR model was similar to that observed in our study with the TAG model for a 5-km buffer around the AQMS. However, Marshall et al.’s study is not directly comparable with ours because they used a larger buffer zone (10 km) and because the LUR and dispersion models incorporated all local sources of pollution, whereas our TAG model did not.

In this study, we focused on women living < 5 km from an AQMS, whereas previous studies on the effects of air pollution on birth weight have included women living > 8 km (5 miles) from a monitor (Basu et al. 2004; Brauer et al. 2008; Parker et al. 2005). Our results indicate that the size of buffer around monitors considered has a major effect on the concordance between models and the estimated association between NO_2_ concentration and birth weight. We obtained higher levels of concordance between the models if we focused on women living within 2 km of a monitor, and higher still for women living within 1 km of a monitor. Associations between NO_2_ levels and birth weight, although not statistically significant at the 5% level, tended to be stronger for the 2-km buffer around the AQMS than for the 5-km buffer (Figure 3). The findings were sometimes less clear for women living within 1 km of an AQMS, and the CIs were slightly larger than for the 2-km buffer, probably because of the small number of subjects. Previous studies with buffers of different sizes gave results similar to ours: Hansen et al. (2008) and Wilhelm and Ritz (2005) found negative associations between fetal growth and levels of exposure to carbon monoxide, coarse particulate matter (≤ 10 μm in aerodynamic diameter), sulfur dioxide, and ozone during pregnancy, as estimated from data from the nearest AQMS, that were stronger for women living within 2 km of a station than for those living up to 14 km away. The choice of the buffer size can probably be seen as a trade-off between bias and variance: The use of smaller buffers decreases sample size (increasing variance) but also probably decreases exposure misclassification (assuming that exposure is better assessed for subjects living closer to an AQMS). However, selection bias may also contribute to the increase in the absolute value of the regression parameter quantifying the association between exposure and birth weight when smaller buffers are considered. Indeed, for associations with third-trimester exposure (but less clearly for first-trimester exposure), the absolute value of the regression parameter also tended to increase as buffer size decreased for the TAG model. This is unlikely to stem from variations in exposure misclassification and might instead be attributed to differences in the selection effects associated with buffers of different sizes.

Most previous studies considering the effects of NO_2_ have reported larger decreases in birth weight for exposure in the first and third trimesters of pregnancy (Bell et al. 2007; Gouveia et al. 2004; Ha et al. 2001; Liu et al. 2007; Mannes et al. 2005; Salam et al. 2005) than in the second trimester or over the entire pregnancy (Ha et al. 2001; Lee et al. 2003; Mannes et al. 2005). We observed a similar pattern in our study. A discussion of the biological relevance of the exposure window or the underlying mechanisms is beyond the scope of this article. Several potential mechanisms by which air pollution may affect fetal growth have been proposed (Kannan et al. 2006; Ritz and Wilhelm 2008; Slama et al. 2008), but none of these mechanisms has been validated.

It is generally difficult to predict the impact of an error in an exposure variable in terms of the potential for bias in the exposure–response relationship (Jurek et al. 2008). However, in the specific case of a Berkson-type error, the power of the study is reduced and CIs are widened, but no bias in linear regression coefficients is expected (Armstrong 2008; Zeger et al. 2000). Berkson-type error (Armstrong 2008) may occur when the exposure is measured at the population level and individual exposures levels vary because of differences in the time windows of exposure or time–activity patterns. The measurement error for the nearest-AQMS approach would be expected to have a Berkson-type error component, because the same proxy exposure is used for all women living in a circular area around a given monitor. The observation that exposure estimates for the nearest-AQMS model were at least as strongly associated with birth weight as those for the TAG model is consistent with the nearest-AQMS model being subject principally to Berkson-type error. Therefore, assuming that the observed association with birth weight was real, exposure misclassification seemed to have little impact on the dose–response relationship. If we accept that the TAG model cannot be seen as a gold standard, exposure mismeasurement seemed to affect both models in similar ways. In a study in Vancouver, Canada, Brauer et al. (2008) found significant negative associations between NO_2_ exposure and fetal growth when they used an AQMS-based approach, but no association when they used an LUR model. They considered women living up to 10 km away from an AQMS, and the AQMS-based model corresponded to an inverse-distance weighting index, taking into account the three closest stations within 50 km.

## Conclusion

Our study indicates that models of exposure to background NO_2_ concentrations based on data from the nearest AQMS may entail large errors in estimated exposure, but that in some instances these errors have little impact on the exposure–birth weight relationship. The amplitude of exposure misclassification in AQMS-based models and of the resulting bias may be limited by restricting the size of the study area around each AQMS considered. Full quantification of the exposure error for each model would require consideration of the temporal and spatial activities of each subject. Our study cannot be interpreted as providing clear evidence that the nearest-AQMS approach yields unbiased estimates of the association between NO_2_ concentrations and fetal growth. This question requires further consideration in other cohorts and in other countries, in which the siting of permanent monitors may follow different rules.

## Figures and Tables

**Table 1:** Characteristics of women living < 5 km away from an AQMS and their associations with NO_2_ levels averaged during pregnancy (*n* = 776).

		Mean (median) NO_2_ level (μg/m^3^)			
Characteristic	*n* (%)	Nearest-AQMS model	*p*-Value^a^	TAG model	*p*-Value^a^
Sex of offspring			0.97		0.28

Male	395 (51)	28.6 (32.3)		23.6 (23.8)	
Female	381 (49)	28.6 (32.5)		23.9 (23.9)	

Gestational duration (weeks)			0.37		0.17

30–36	48 (6)	30.2 (33.4)		24.7 (23.1)	
37–38	151 (20)	29.1 (32.6)		24.3 (24.1)	
39–40	407 (52)	28.1 (32.2)		23.4 (23.6)	
≥ 41	170 (22)	29.2 (32.8)		23.8 (24.3)	

Birth order			0.71		0.14

First birth	367 (47)	28.8 (33.4)		23.9 (23.9)	
Second birth	263 (34)	28.7 (31.7)		23.9 (24.0)	
Third birth or more	145 (19)	28.0 (32.2)		23.0 (23.1)	
Missing value	1				

Trimester of conception of the child			< 10^−4^		< 10^−4^

January–March	167 (21)	25.7 (25.3)		21.5 (21.9)	
April–June	184 (24)	29.1 (33.6)		23.5 (24.0)	
July–September	226 (29)	31.2 (35.2)		25.9 (25.7)	
October–December	199 (26)	27.7 (31.3)		23.3 (23.5)	

Maternal age at conception (years)			< 10^−2^		< 10^−2^

< 25	187 (24)	26.7 (26.3)		22.8 (22.7)	
25–29	289 (37)	30.0 (33.8)		24.3 (24.3)	
30–34	203 (26)	28.7 (32.1)		24.2 (24.0)	
≥ 35	97 (13)	27.9 (32.3)		22.9 (23.4)	

Maternal height (cm)			0.64		0.44

< 160	188 (24)	28.3 (32.0)		23.4 (24.0)	
160–169	460 (60)	28.6 (32.7)		23.8 (23.8)	
≥ 170	121 (16)	29.4 (33.1)		24.2 (24.2)	
Missing value	7				

Maternal prepregnancy weight (kg)			0.33		0.46

< 50	83 (11)	27.7 (28.8)		24.3 (24.1)	
50–59	333 (43)	28.6 (32.3)		23.8 (23.8)	
60–69	211 (27)	29.4 (33.5)		23.8 (24.0)	
70–79	87 (11)	29.0 (33.0)		23.6 (23.8)	
≥ 80	60 (8)	26.6 (25.9)		22.7 (22.0)	
Missing value	2				

Body mass index before pregnancy (kg/m^2^)			0.39		0.07

< 18.5	82 (11)	29.6 (34.3)		25.0 (24.7)	
18.5–24.9	512 (67)	28.5 (32.1)		23.8 (23.9)	
25–29.9	111 (14)	29.4 (33.7)		23.3 (23.4)	
≥ 30	62 (8)	27.1 (30.6)		23.0 (22.4)	
Missing value	9				

Center			< 10^−4^		< 10^−4^

Poitiers	316 (41)	24.9 (18.8)		20.3 (19.2)	
Nancy	460 (59)	31.2 (34.4)		26.1 (25.7)	

Maternal age at end of education (years)			0.02		< 10^−3^

≤ 16	52 (7)	29.6 (33.1)		24.0 (23.6)	
17–18	104 (13)	27.0 (29.6)		22.2 (21.9)	
19–20	124 (16)	27.1 (29.1)		23.2 (23.0)	
21–22	165 (21)	27.9 (30.0)		23.3 (23.5)	
23–24	174 (22)	29.3 (33.1)		24.5 (24.6)	
≥ 25	157 (20)	30.6 (34.5)		24.7 (24.6)	

Maternal active smoking (second trimester)			0.45		0.30

No	641 (83)	28.8 (32.7)		23.8 (24.0)	
Yes	133 (17)	28.1 (32.0)		23.3 (22.8)	
Missing value	2				

Maternal passive smoking (second trimester)			0.48		0.53

No	507 (66)	28.5 (32.1)		23.7 (23.9)	
Yes	264 (34)	29.0 (33.3)		23.9 (23.6)	
Missing value	5				

a *p*-Value comparing model-specific exposure estimates between categories (Student test for dichotomous variables) or among categories (Fisher’s analysis of variance for variables with more than two categories). Tests were performed without including missing data as a separate category.

**Table 2:** Maternal exposure to NO_2_ (μg/m^3^) and concordance between NO_2_ levels [mean ± SD (5th, 50th, 95th percentiles)] estimated by the nearest-AQMS model and the TAG model, for various exposure windows and buffer sizes considered around AQMSs.

Area exposure window	Nearest-AQMS model (5-km buffer)		TAG model (5-km buffer)			Between-model agreement											
						Distance^a^ < 5 km				Distance^a^ < 2 km				Distance^a^ < 1 km			
	*n*	NO_2_ levels	*n*	NO_2_ levels	*p*-Value^b^	*n*	*r*	*c*	κ	*n*	*r*	*c*	κ	*n*	*r*	*c*	κ
Both areas																	
First trimester	770	28.8 ± 10.8 (11.3, 30.1, 43.6)	773	23.7 ± 6.2 (13.6, 23.0, 34.6)	10^−4^	767	0.67	61	0.41	429	0.70	62	0.43	158	0.83	75	0.63
Second trimester	771	29.0 ± 10.9 (11.5, 30.0, 43.9)	770	24.1 ± 6.5 (13.6, 23.6, 34.4)	10^−4^	766	0.69	60	0.40	426	0.72	58	0.37	156	0.82	73	0.60
Third trimester	770	28.1 ± 11.1 (10.4, 29.4, 44.2)	772	23.3 ± 6.8 (12.5, 22.8, 34.7)	10^−4^	767	0.74	63	0.44	428	0.79	68	0.52	155	0.87	79	0.68
Whole pregnancy	776	28.6 ± 10.0 (13.3, 32.4, 41.8)	770	23.7 ± 5.0 (16.1, 23.8, 32.3)	10^−4^	770	0.65	63	0.44	428	0.70	64	0.46	157	0.85	73	0.59

Poitiers area																	
First trimester	310	25.6 ± 11.9 ( 9.3, 21.6, 43.0)	316	20.9 ± 6.3 (12.0, 20.4, 35.8)	< 10^−3^	310	0.61	59	0.38	181	0.65	57	0.36	75	0.89	83	0.74
Second trimester	311	25.2 ± 11.6 (10.1, 22.2, 42.7)	315	20.4 ± 6.1 (11.8, 19.9, 32.0)	10^−4^	311	0.61	56	0.34	179	0.65	57	0.36	74	0.83	63	0.45
Third trimester	310	23.9 ± 11.3 ( 8.5, 21.7, 42.0)	315	19.5 ± 6.3 (11.5, 19.0, 30.8)	10^−4^	310	0.66	62	0.43	179	0.72	67	0.51	73	0.86	78	0.67
Whole pregnancy	316	24.9 ± 10.6 (12.4, 18.8, 40.5)	316	20.3 ± 4.7 (14.7, 19.2, 30.0)	0.12	316	0.55	56	0.34	181	0.62	58	0.37	75	0.87	68	0.52

Nancy area																	
First trimester	460	31.0 ± 9.5 (13.6, 31.3, 44.1)	457	25.7 ± 5.2 (17.9, 25.5, 34.6)	10^−4^	457	0.67	55	0.32	248	0.69	58	0.36	83	0.72	59	0.39
Second trimester	460	31.6 ± 9.6 (14.1, 32.0, 44.4)	455	26.7 ± 5.5 (18.5, 26.6, 35.6)	10^−4^	455	0.70	58	0.37	247	0.73	65	0.48	82	0.74	66	0.49
Third trimester	460	30.9 ± 10.0 (13.5, 31.4, 45.0)	457	26.0 ± 5.8 (17.5, 25.7, 36.2)	10^−4^	457	0.74	61	0.41	249	0.78	67	0.51	82	0.82	76	0.63
Whole pregnancy	460	31.2 ± 8.7 (16.9, 34.4, 42.4)	454	26.1 ± 3.7 (20.8, 25.7, 32.8)	10^−4^	454	0.66	64	0.46	247	0.69	64	0.47	82	0.66	71	0.56

Abbreviations: *r*, Pearson correlation coefficient; *c*, concordance percentage (based on NO_2_ levels categorized in tertiles); κ, kappa coefficient (based on NO_2_ levels categorized in tertiles).

a Maximal distance between home address and the nearest AQMS (buffer size).

b *p*-Value of Kruskal–Wallis rank test comparing the exposure levels from the two models.

**Table 3:** Variance component (%) of NO_2_ exposure levels estimated by the nearest-AQMS model and by the TAG model for various exposure windows and buffer sizes considered around AQMSs.

	Distance < 5 km (*n* = 681)				Distance < 2 km (*n* = 383)				Distance < 1 km (*n* = 146)			
	Nearest-AQMS model		TAG model		Nearest-AQMS model		TAG model		Nearest-AQMS model		TAG model	
Exposure window	Spatial	Temporal	Spatial	Temporal	Spatial	Temporal	Spatial	Temporal	Spatial	Temporal	Spatial	Temporal
First trimester	82	21	61	52	79	22	55	57	84	25	56	49
Second trimester	82	20	55	46	79	21	53	52	83	21	58	44
Third trimester	78	21	47	46	76	21	43	52	80	24	52	48
Pregnancy	95	7	92	14	91	8	92	17	97	9	92	13

The sum of variance components is > 100% because the data are not balanced as in experimental plans (i.e., the covariance is not null).
